# Loureirin B Exerts its Immunosuppressive Effects by Inhibiting STIM1/Orai1 and K_V_1.3 Channels

**DOI:** 10.3389/fphar.2021.685092

**Published:** 2021-06-25

**Authors:** Shujuan Shi, Qianru Zhao, Caihua Ke, Siru Long, Feng Zhang, Xu Zhang, Yi Li, Xinqiao Liu, Hongzhen Hu, Shijin Yin

**Affiliations:** ^1^ Department of Chemical Biology, School of Pharmaceutical Sciences, South-Central University for Nationalities, Wuhan, China; ^2^ Department of Anesthesiology, the Center for the Study of Itch & Sensory Disorders, Washington University School of Medicine, St. Louis, MO, United States

**Keywords:** LrB, Kv1.3, Jurkat T cell, CRISPR/ Cas9, STIM1/Orai1, Ca^2+^ influx, IL-2

## Abstract

Loureirin B (LrB) is a constituent extracted from traditional Chinese medicine Resina Draconis. It has broad biological functions and an impressive immunosuppressive effect that has been supported by numerous studies. However, the molecular mechanisms underlying Loureirin B-induced immune suppression are not fully understood. We previously reported that Loureirin B inhibited K_V_1.3 channel, calcium ion (Ca^2+^) influx, and interleukin-2 (IL-2) secretion in Jurkat T cells. In this study, we applied CRISPR/Cas9 to edit K_V_1.3 coding gene KCNA3 and successfully generated a K_V_1.3 knockout (KO) cell model to determine whether K_V_1.3 KO was sufficient to block the Loureirin B-induced immunosuppressive effect. Surprisingly, we showed that Loureirin B could still inhibit Ca^2+^ influx and IL-2 secretion in the Jurkat T cells in the absence of K_V_1.3 although KO K_V_1.3 reduced about 50% of Ca^2+^ influx and 90% IL-2 secretion compared with that in the wild type cells. Further experiments showed that Loureirin B directly inhibited STIM1/Orai1 channel in a dose-dependent manner. Our results suggest that Loureirin B inhibits Ca^2+^ influx and IL-2 secretion in Jurkat T cells by inhibiting both K_V_1.3 and STIM1/Orai1 channels. These studies also revealed an additional molecular target for Loureirin B-induced immunosuppressive effect, which makes it a promising leading compound for treating autoimmune diseases.

## Introduction

Loureirin B (LrB) is a Resina Draconis (RD)-derived flavonoid and a traditional Chinese medicine bearing multifaceted effects on numerous diseases (Fan et al., 2014; Bai et al., 2015). LrB was reported to be a plasminogen activator inhibitor-1 (PAI-1) that promoted blood circulation and reduced the size of arterial thrombus (Jiang et al., 2017). LrB alone or combined with other RD constituents inhibited voltage-gated sodium (Na_V_) channel, transient receptor potential vanilloid 1 channel, and acid-sensing ion channel in dorsal root ganglion (DRG) neurons and ameliorated inflammatory pain (Chen et al., 2018; Wan et al., 2019). Besides its analgesic effect, LrB and RD also possess promising immunosuppressive effects. The ethylacetated RD inhibited inflammatory responses in vascular smooth muscle cells and macrophages by suppressing ROS production (Heo et al., 2010). A recent study showed that LrB could reduce the severity of inflammation in Crohn’s disease *via* inhibiting the expression levels of inflammatory cytokines interleukin-1 (IL-1), IL-6, and tumor necrosis factor-alpha (TNF-α) (Sun et al., 2020). However, the mechanisms behind LrB-induced immune suppression have not been fully elucidated.

Ion channels are critically involved in regulating proliferation and apoptosis of lymphocytes (Cahalan and Chandy, 2009). Calcium signaling plays a pivotal role in linking ion channels and numerous functions of T lymphocytes (Feske et al., 2015). When an antigen stimulates a T cell and binds to a T cell receptor (TCR), the downstream phospholipase C gamma (PLC*γ*) will hydrolyze PIP_2_ into DAG and inositol triphosphate (IP_3_) (Zhong et al., 2016; Zhu et al., 2017). The newly synthesized IP_3_ binds to IP_3_ receptor (IP_3_R) on endoplasmic reticulum (ER) membrane and depletes calcium ion (Ca^2+^) stored in ER pool (Zhong et al., 2016; Cao et al., 2019). Another ER membrane protein - stromal interaction molecule (STIM) - could sense Ca^2+^ depletion in ER and change its own conformation to contact and subsequently open a Ca^2+^ channel - Orai channel on the plasma membrane (Lewis, 2020). This process is named Store-operated Calcium Entry (SOCE) and results in Ca^2+^ influx which ultimately activates the downstream signaling pathways such as CaM-CaN-NFATc1 and NFκB to produce inflammatory cytokines (Feske et al., 2012; Trebak and Kinet, 2019). During these processes, the STIM1/Orai1 complex - also called calcium release-activated calcium (CRAC) channel - is the “major player” to trigger immune responses of T cells, while other ion channels, such as potassium ion (K^+^) channel, could modulate calcium signals by changing the membrane potential of T cells and providing a driving force for Ca^2+^ entry (Cahalan and Chandy, 2009).

K_V_1.3 is a predominantly expressed K^+^ channel in T cells (Nicolaou et al., 2009) and regulates immune responses stimulated by antigens and cell volume of T cells (Nicolaou et al., 2009; Bobak et al., 2011). Pharmacological blockade of K_V_1.3 in myelin basic protein - specific encephalitogenic T cell line inhibited cytokine secretion through reducing Ca^2+^ influx, which improved encephalomyelitis (EAE) symptoms in rats (Beeton et al., 2001). K_V_1.3 blockade also suppressed the activation and motility of T_EM_ (memory effector T) cells and delayed-type hypersensitivity in rats (Matheu et al., 2008). Surprisingly, although K_V_1.3 plays an important role in T cells, K_V_1.3 knockout (KO) mice lived normally and their immune systems showed no impairment (Koni et al., 2003). The expression level of chloride ion (Cl^−^) channels was found to have increased 10-fold in T lymphocytes from these KO animals, which could compensate for the function of K_V_1.3 to sustain a normal membrane potential and did not affect proliferation or activation of T cells (Koni et al., 2003). Another report studied the effects of K_V_1.3 deletion in the EAE model and found that the number of activated CD4^+^ T cells and secretion of IFN-γ and IL-17 in K_V_1.3 KO mice were all decreased significantly (Gocke et al., 2012). A recent study showed that T helper (Th) cells isolated from the K_V_1.3 KO mice developed into a novel type of Th cell which differed in gene expression and functions compared with Th cell in wild type animals when stimulated with Myelin oligodendrocyte glycoprotein (MOG) (Grishkan et al., 2015). These inconsistent results in K_V_1.3 KO mice suggest that selective K_V_1.3 deletion in specific lymphocyte type might help to pinpoint the mechanisms underlying K_V_1.3-mediated regulation of immune responses in healthy or pathological organisms.

We previously used Jurkat T cell as a model to study the effects of LrB and reported that LrB inhibited K_V_1.3 currents, Ca^2+^ influx, and IL-2 release in a dose-dependent manner (Yin et al., 2014). In this study, we applied CRISPR/Cas9 system to knock out KCNA3 gene to explore whether K_V_1.3 deletion could block effects of LrB on Ca^2+^ influx and cytokine secretion. We showed that KO K_V_1.3 in Jurkat T cell decreased but did not abolish the effects of LrB on Ca^2+^ influx and IL-2 secretion. We further demonstrated that LrB could decrease Ca^2+^ influx and IL-2 secretion through inhibiting STIM1/Orai1 channels in the absence of K_V_1.3. These findings suggest that LrB has an immunosuppressive effect by inhibiting both K_V_1.3 and STIM1/Orai1 channels.

## Materials and Methods

### Drugs

LrB was synthesized by our group (Supplementary Scheme S1) and dissolved in DMSO. K_V_1.3 inhibitor ADWX-1 was purchased from More Biotechnology Co. Ltd. (Cat. MPK-001A, Wuhan, China) and dissolved in double-distilled water. Agonist of T cell - Concanavalin A (ConA) was purchased from MP Biomedicals (Cat. 195,283, Santa Ana, CA, United States) and dissolved in 1x Phosphate Buffer Saline (PBS). Calcium-ATPase inhibitor Cyclopiazonic Acid (CPA) was purchased from Sigma-Aldrich (Cat. C1530, St.Louis, MO, United States) and dissolved in DMSO. SK_Ca_ inhibitor Scytx was purchased from More Biotechnology Co. Ltd. (Cat. MPK-001C, Wuhan, China).

### Cell Culture

Jurkat T cell line was bought from National Platform of Cell Line Resource for Sci-Tech (Wuhan, China). Wild type and K_V_1.3 knockout Jurkat T cells were suspended in culture medium which consisted of Roswell Park Memorial Institute (RPMI) 1,640 basic (Cat. C11875500, Gibco, NY, United States) supplemented with 10% fetal bovine serum (FBS) (Cat. 40130ES76, YESEN, Shanghai, China) and 1% Penicillin-Streptomycin (Cat. 15070063, Gibco, NY, United States). Cell density was counted using Cellometer K2 (Nexcelom, San Diego, CA, United States) and suspended cells were seeded in 6-well plate with 1 × 10^6^ cells/well in 2 ml culture medium under 5% CO_2_ and 37°C. After being pretreated with 0.01, 0.1, or 1 μM LrB for 1 h, 10 μg/ml ConA was added in the culture medium as stimulus group, and the same volume of 1x PBS was added in control group. After incubating with ConA or PBS for 24 h, the culture medium was harvested in Eppendorf (Ep) tube for ELISA assay (Cat. DY202-05, R&D, Minneapolis, MN, United States) to test IL-2 secretion by Jurkat T cells with or without ConA stimulation.

### sgRNA Design and Plasmid Construct

sgRNA was designed to target the first exon (exon1) of KCNA3 gene, which consists of the functional domain of K_V_1.3 subunit. The target DNA sequence was submitted to http://crispr.mit.edu/website and two sgRNA sequences with the highest editing efficiency scores were selected and synthesized by Tskingke Company (Peking, China). Sequences of DNA fragments expressing sgRNAs CTA​CCC​CGC​CTC​GAC​GTC​GC (sgRNA1) and GAG​ATC​CGC​TTC​TAC​CAG​CT (sgRNA2) are used in our study (Supplementary Figures S1A,B). The pX458 plasmid is a gift from Lu Xue lab (School of Life Sciences, Central-South University for Nationalities, Wuhan, China) and used to express SpCas9 and sgRNA simultaneously to edit the target genes. This plasmid expresses GFP as a fluorescence reporter to indicate vector transfection of the cells. The plasmid was digested with Fast Digest BbsⅠ enzyme (Cat. ER1011, Thermo Scientific, Waltham, MA, United States) and linked with synthesized sgRNA fragments by T4 ligase (Cat. D2011A, Kyoto, Takara, Japan). The sgRNA sequence insertion in plasmid was confirmed by gene sequencing (Supplementary Figure S1A,B).

### Electroporation and Cell Sorting

Cultured Jurkat T cells were transfected with the constructed plasmids using a CTX-1500A EX Electroporator (Celetrix Biotechnologies, Manassas, VA, United States). Cultured Jurkat T cells were suspended into single cells and sorted with Fluorescence Activated Cell Sorter (FACS) (MA900, Sony, Tokyo, Japan) 48 h after transfection. The single cells expressing GFP were selected and seeded in a 96-well plate and passed to a 24-well and then a 6-well plate to amplify the single colony. Genomes and proteins were extracted from each colony for confirmation of K_V_1.3 KO.

### Confirmation of Gene Editing Efficiency

Before cell sorting by FACS, Jurkat T cells transfected with the constructed plasmids were tested for editing efficiency first. Genomes in different groups were extracted using Genome Extraction kit (Cat. 7E491E0, Vazyme, Suzhou, China) and the target sequence was amplified with Polymerase Chain Reaction (PCR) from the genome. The PCR primers were FP: GTC​ATC​AAC​ATC​TCC​GGG​CT, RP: TAC​TCG​AAG​AGC​AGC​CAC​AC. Products of PCR were used for T7EN1 digestion and DNA gel first. Gray analysis of the digested fragments compared with the whole quantities of the PCR products was used to calculate the editing efficiency of CRISPR/Cas9. After we obtained the single cell colony, the target sequence was amplified with PCR and ligated into commercial pMD™19 T vector (Cat. 6,013, Kyoto, Takara, Japan) to check the mutation of each single colony edited by PX458-sgRNA1 or PX458-sgRNA2.

### Western Blot

Cultured cells were collected by centrifugation at 6,000 rpm for 5 min and washed with ice cold 1x PBS twice. Cells were lysed with Radio Immunoprecipitation Assay (RIPA) solution (Cat. P0013B, Beyotime, Peking, China) for 30 min. The lysis solution was centrifuged again at 12,000 rpm under 4°C for 15 min and the supernatant was collected and mixed with 2x Sodium dodecyl sulfate (SDS) loading buffer and boiled for 5 min. Before loading, the total protein concentration of each group was determined by BCA kit and scanned with a microplate photospectrometer (SPARK 10M, Tecan, Hombrechtikon, Swiss). Proteins were loaded based on the total protein concentration of each group to assure equal quantities per lane. Proteins were separated on a 10% SDS-polyacrylamide gel and transferred to Polyvinylidene fluoride (PVDF) membranes (Millipore, Billerica, MA). The membranes were blocked with 10% nonfat milk and incubated at 4°C overnight with the following antibodies: rabbit polyclonal antibody against K_V_1.3 (1:1,000, Cat. APC101; alomone labs, Jerusalem, Israel) and rabbit polyclonal antibody against *β*-actin (1:1,000, Cat. AC006; Abclonal, Boston, United States). After washing in Tris-Buffered Saline (TBS) with 0.3% Tween three times for 45 min, the membranes were incubated with HRP-conjugated goat anti-rabbit IgG (1:10,000, Cat. AS014; Abclonal, Boston, United States) for 2 h at room temperature. Chemiluminescent signals were generated using a Super Signal West Pico trial kit (Pierce Protein Biology, Thermo Fisher Scientific, Waltham, MA, United States) and detected using the ChemiDoc XRS System (Bio-Rad, Hercules, CA, United States). Image Lab software (Bio-Rad, Hercules, CA, United States) was used for background subtraction and quantification of immunoblotting data.

### Electrophysiology Recording

Whole-cell patch-clamp recordings were performed using an EPC9 amplifier (HEKA Elektronik, Lambre-cht/Pfalz, Germany) at room temperature (22–24°C). Pipettes pulled from borosilicate glass (Cat. BF 150-86-10, Sutter Instrument Co., Novato, CA, United States) had resistances of 2–4 MΩ when filled with the internal solution. The internal pipette solution for recording voltage-gated potassium (K_V_) currents contained KCl 140 mM, MgCl_2_ 1 mM, EGTA 1 mM, Na_2_ATP 3 mM, and HEPES 10 mM (pH 7.3 with KOH). For recording CRAC, currents contained Cs Methanesulfonate 120 mM, MgCl_2_ 10 mM, EGTA 4 mM, CaCl_2_ 2 mM, and HEPES 10 mM (pH 7.3 with CsOH). The formula of internal pipette solution for recording small conductance Ca^2+^-activated K^+^ channels (SK_Ca_) currents was calculated by Winmaxc software to guarantee that the concentration of free Ca^2+^ in cytoplasm was kept at 2 μM and contained KCl 140 mM, HEPES 10 mM, HEDTA 5 mM, and CaCl_2_ 2 mM (pH 7.2 with KOH). The external solution for recording K_V_ currents contained KCl 5 mM, NaCl 140 mM, HEPES 10 mM, CaCl_2_ 2 mM, MgCl_2_ 1 mM, and d-Glucose 10 mM (pH 7.4 with NaOH). For recording CRAC currents, the external solution contained NaCl or NaGlu 140 mM, KCl 5 mM, HEPES 10 mM, or CaCl_2_ 2.5 mM (pH 7.3 with NaOH). For recording SK_Ca_ currents the external solution contained KCl 140 mM, MgCl_2_ 1 mM, Glucose 10 mM, CaCl_2_ 2 mM, and HEPES 10 mM (pH 7.4 with KOH). K_V_ currents were elicited by +50 mV, 400 ms depolarizing pulse from the holding potential of −60 mV every 20 s. CRAC currents were elicited by +10 mV, 100 ms depolarizing ramp from the holding potential of -60 mV every 10 s, and SK_Ca_ currents were elicited by from −100 mV to +100 mV for 200 ms depolarizing ramp from the holding potential of 0 mV every 10 s. Using IGOR (WaveMetrics, Lake Oswego, OR, United States) software, concentration–response relationships were fitted according to modified Hill equation: I_toxin_/I_control_ = 1/1 + ([drug]/IC_50_), where I is the steady-state current and [drug] is the concentration of LrB. The parameter to be fitted was concentration of half-maximal effect (IC_50_).

### IL-2 Secretion

IL-2 secretion from Jurkat T cells was measured using an ELISA kit (Cat. DY202-05, R&D System, Minneapolis, MN, United States) following manufacturer’s instructions. The goodness of fit for the ELISA kit was between 10 pg/ml to 1,000 pg/ml. Cells were centrifuged at 1,500 rpm for 10 min, and the supernatants were collected to measure IL-2 concentrations. Reactions were performed in 96-well plates coated with the capture antibody and stopped with 1 M phosphoric acid. Absorbance was measured at 450 nm. Each experiment was repeated at least three times in duplicate.

### Calcium Imaging

Jurkat T cells were loaded with 4 μM Fura-2 AM (Cat.40702ES50, YEASEN, Shanghai, China) for 60 min at 37 °C. Cells were then washed three times and incubated in Hank’s Balanced Salt Solution (HBSS) for 30 min at room temperature before use. Fluorescence at 340 and 380 nm excitation wavelengths was recorded on an inverted Nikon Ts2R microscope (Tokyo, Japan) equipped with 340, 360, and 380 nm excitation filter wheels using NIS-Elements imaging software (Nikon). 510 nm Fura-2 emission fluorescence ratios (F340/F380) reflect changes in intracellular Ca^2+^ concentration ([Ca^2+^]_i_) upon stimulation. Data were obtained from 100 to 250 cells in time-lapse images from each coverslip. To make sure that Fura-2 loading was the same in individual cells, we selected the cells with the same baseline level of fluorescence intensity at the beginning of the calcium imaging experiment.

### Statistics Analysis

All data are presented as mean ± SEM for independent observations. Statistical analysis of differences between groups was carried out using one-way ANOVA combined with Turkey’s post-hoc test. *p* < 0.05 was considered significantly different.

## Results

### CRISPR/Cas9-Mediated K_V_1.3 Knockout in Jurkat T Cells

We constructed the pX458-sgRNA1 and pX458-sgRNA2 plasmids to express Cas9 and sgRNA simultaneously and confirmed the insertion of sgRNA with sequencing (Supplementary Figure S1A,B). The Jurkat T cells were transfected by electroporation with the constructed plasmids that also express GFP as a reporter (Supplementary Figure S1C). The target sequence was amplified with PCR and digested by T7EN1 enzyme to confirm editing efficacy. If the target sequence had been edited by CRISPR/Cas9, the hybridized double strand DNA would be mismatched and form a neck and ring structure. T7EN1 enzyme could recognize the mismatched DNA structure and cut it into small fragments. DNA gel of the digestion products showed that the control group only had one single band, which is consistent with the molecular weight of the target sequence, while sgRNA1 and the sgRNA2 editing groups both had bands smaller than the target sequence. These bands matched the fragments cut by T7EN1 (Supplementary Figure S1D). The editing efficiencies of sgRNA1 and sgRNA2 calculated according to the gray analysis of T7EN1 digestion were 25 and 34%, respectively.

**FIGURE 1:**
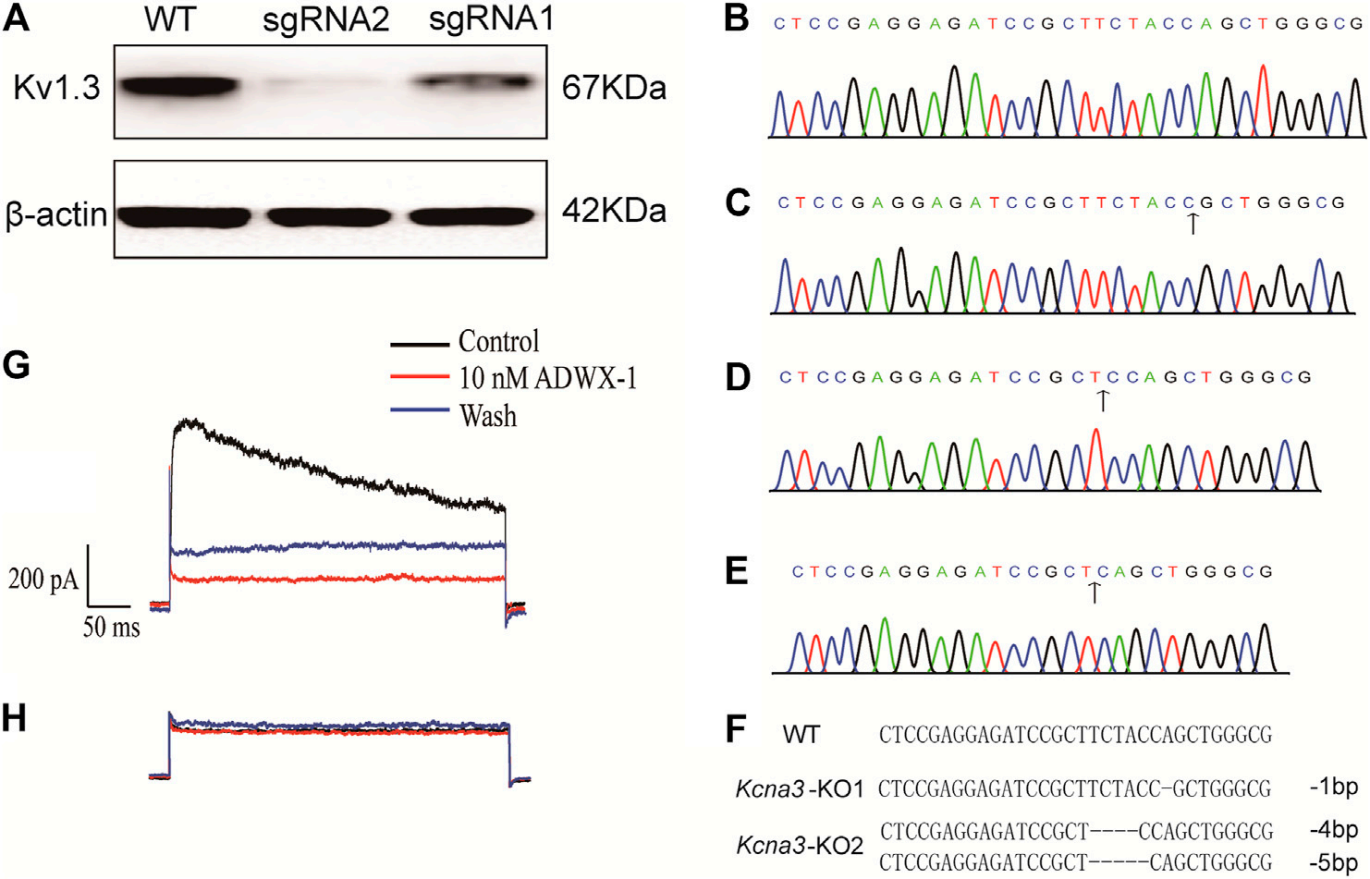
Confirmation of Kv1.3 KO in Jurkat T cells. **(A)** Western Blot for K_V_1.3 expression in wild type and Kv1.3 KO cells (*n* = 3); **(B)** Sequencing of target gene in wild type Jurkat T cells; **(C)** Sequencing of target gene in KO-1 colony which has one bp nucleotide loss compared with wild type, the black arrow indicates the start point of CRISPR/Cas9 editing; **(D,E)** KO-2 colony has four and five bp nucleotide loss respectively, the black arrow indicates the start point of CRISPR/Cas9 editing; **(F)** Alignment of the wild type, KO-1 and KO-2 Jurkat T cell colonies; **(G)** Membrane current of a wild type Jurkat T cell was irreversibly inhibited by 10 nM ADWX-1 (*n* = 3); **(H)** Membrane current of a Kv1.3 knockout Jurkat T cell was nearly abolished and the remaining residual current was not further inhibited by ADWX-1 (*n* = 3).

To obtain the KCNA3 knock out Jurkat T cell, we used FACS to select GFP-expressing cells which had been transfected with pX458-sgRNA plasmid and edited by CRISPR/Cas9 system. Single cells were then seeded in a 96-well plate and cultured for colony expansion. Proliferated cells were harvested and lysed with RIPA solution to extract proteins to examine the expression of K_V_1.3 using Western Blot. We found that cell colonies edited either by sgRNA1 or sgRNA2 expressed much less K_V_1.3 than the control group (Figure 1A). K_V_1.3 expression in the colony edited by sgRNA2 was lower than that by sgRNA1, and almost completely knocked out by CRISPR/Cas9 editing (Figure 1A). We therefore used the sgRNA2-edited cells for further experiments.

The genome of sgRNA2-edited cells was extracted and the target sequence of K_V_1.3 was amplified with PCR. The PCR product was sequenced and nucleotide deletion started around the recognition site of sgRNA, which indicates Indel mutations from this site. To further confirm the mutation of the targeted sequence, the PCR fragments were ligated into pMD™19 T vector and sequenced again. We found that one cell colony (KO-1) lost one bp (Figures 1C,F) compared with the wild type group (Figures 1B,F). Another cell colony (KO-2) lost four and five bp (Figures 1D–F), respectively. We compared the sequences of KO-1 and KO-2 with wild type sequence and confirmed that these two colonies both had frameshift mutations starting from the sgRNA guided site and changed the open reading frame of KCNA3, thus changing the normal K_V_1.3 translation.

### K_V_1.3 Current Elimination in Jurkat T Cell After CRISPR/Cas9 Editing

After confirmation of KCNA3 editing efficiency and K_V_1.3 expression deletion, we studied the electrophysiology properties of the K_V_1.3 KO cells and compared the changes in membrane currents with wild type cells. Previous studies showed that K_V_1.3 and type II SK_Ca_ (K_Ca_2.2) channels were the two main types of K^+^ channels expressed in Jurkat T (Cahalan and Chandy, 2009; Valle-Reyes et al., 2018). To determine whether KO K_V_1.3 eliminates the function of K_V_1.3 in Jurkat T cell, we used whole-cell patch-clamp recording to measure K^+^ currents in Jurkat T cells. The holding potential was set as −60mV and depolarized to +50 mV for 400 ms and a Ca^2+^-free external solution with added tetrodotoxin (TTX) to block Na_V_ currents was used to record K^+^ currents. A large outward current was recorded in wild type Jurkat T cells, which was blocked by the K_V_1.3 inhibitor ADWX-1 (Figure 1G). The outward current amplitude was much smaller in the K_V_1.3 KO Jurkat T cells and was resistant to 10 nM ADWX-1 (Figure 1H), suggesting that Jurkat T cells edited by CRISPR/Cas9 system completely lost K_V_1.3 conducted current.

### LrB-Induced Inhibiton of IL-2 Secretion in Jurkat T Cells

**FIGURE 2:**
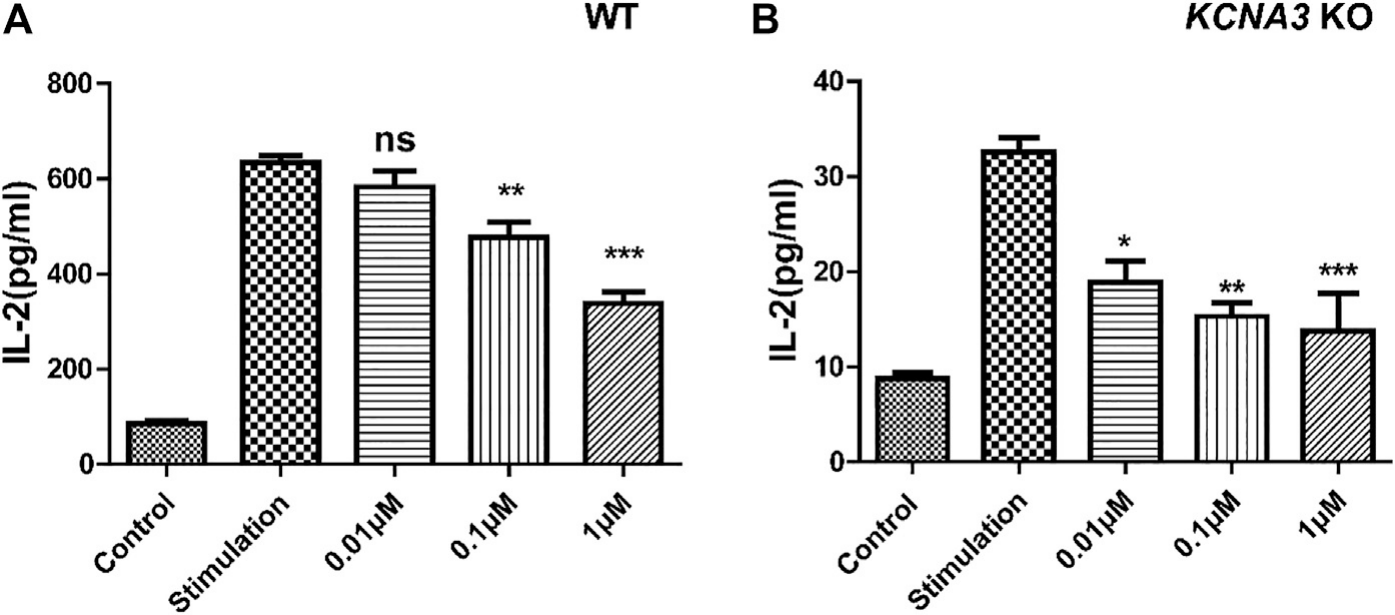
Effects of Kv1.3 KO on IL-2 secretion. **(A)** Changes of IL-2 secretion in wild type Jurkat T cells under control, ConA stimulation, and LrB treatment with different concentrations (*n* = 5); **(B)** Changes of IL-2 secretion in the Kv1.3 KO Jurkat T cells under control, ConA stimulation, and LrB treatment with different concentrations (*n* = 5). Data were presented as mean ± SEM. Statistical analysis of differences between groups was carried out using one-way ANOVA combined with Turkey’s post-hoc test. ***, *p* < 0.001.

Concanavalin A (ConA) is a phytohemagglutinin activating T cells and increasing K_V_1.3 expression on T cell membrane (Yin et al., 2014; Yabuuchi et al., 2017). ConA also induced IL-2 release through a Ca^2+^-dependent pathway in Jurkat T cells (Sharma et al., 2019). We therefore used ConA as a cytokine stimulant. The control group and K_V_1.3 KO Jurkat T cells under the same culture conditions were pretreated with 0.01, 0.1, or 1 μM LrB separately for 1 h, then 10 ug/mL ConA was added in the culture medium to activate Jurkat T cells. 24 h after ConA incubation, the supernatant of culture medium was collected and tested for IL-2 concentration using an ELISA kit. The quantity of IL-2 secreted by the K_V_1.3 KO group (8.73 ± 0.66 pg/ml) without any treatment was 10-fold lower compared with the wild type group (85.00 ± 6.46 pg/ml) (Figures 2A,B). Surprisingly, LrB in a relatively low concentration still significantly inhibited IL-2 secretion although most IL-2 release was blocked by KO K_V_1.3 (Figure 2B). Moreover, the inhibitory effect of LrB on IL-2 secretion in the wild type Jurkat T cells was dose-dependent (*p* < 0.001) in both wild type and K_V_1.3 KO cells treated with 0.01, 0.1, or 1 μM LrB. Therefore, LrB could inhibit IL-2 release in the absence of K_V_1.3.

### Decreased Ca^2+^ Influx in K_V_1.3 KO Jurkat T Cells

**FIGURE 3:**
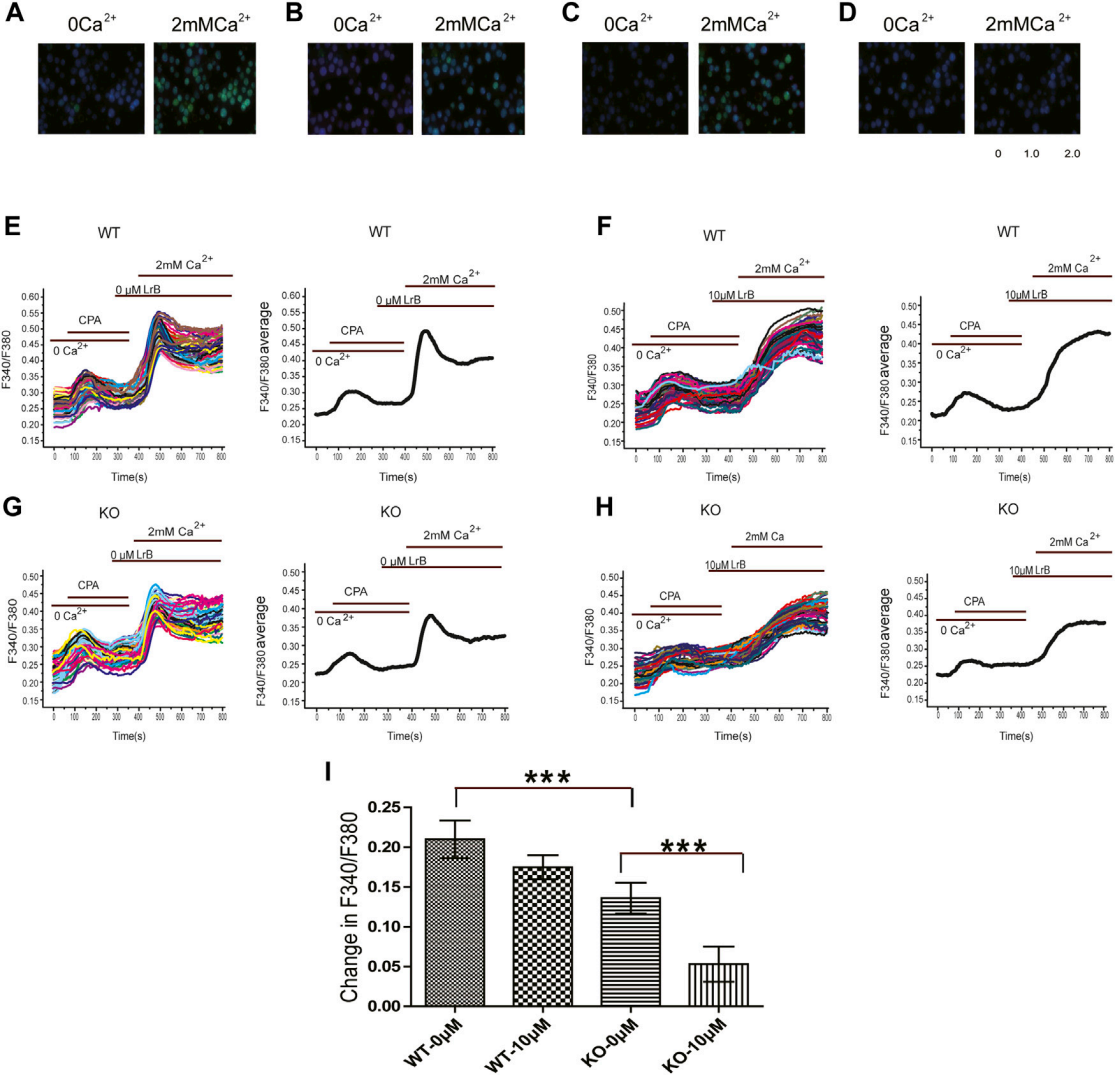
Effects of LrB on [Ca^2+^]_i_ in Jurkat T cells. **(A)** [Ca^2+^]_i_ responses of wild type Jurkat T cells before **(left)** and after **(right)** 2 mM Ca^2+^perfusion; **(B)** [Ca^2+^]_i_ responses of wild type Jurkat T cells pretreated with 10 μM LrB before **(left)** and after **(right)** 2 mM Ca^2+^perfusion; **(C)** [Ca^2+^]_i_ responses of the Kv1.3 KO Jurkat T cells before **(left)** and after **(right)** 2 mM Ca^2+^perfusion; **(D)** [Ca^2+^]_i_ responses of the Kv1.3 KO Jurkat T cells pretreated with 10 μM LrB before **(left)** and after **(right)** 2 mM Ca^2+^perfusion; **(E,F)** Representative traces of F340/F380 ratio show [Ca^2+^]_i_ in wild type Jurkat T cells treated with and without LrB; the first small peak was caused by 10 μM CPA depletion of ER Ca^2+^store, the second peak was caused by 2 mM Ca^2+^perfusion; **(G,H)**. Representative traces of F340/F380 ratio in the Kv1.3 KO Jurkat T cells treated with and without LrB; **(I)** Statistic analysis of changes in F340/F380 in the wild type and Kv1.3 KO cells after 2 mM Ca^2+^perfusion between control and LrB treatment groups (*n* = 3, 100–250 cells were selected each batch). Data were presented as mean ± SEM. Statistical analysis of differences between groups was carried out using one-way ANOVA combined with Turkey’s post-hoc test. ***, *p* < 0.001.

K_V_1.3 conducts an outward K current and hyperpolarizes the membrane potential, which provides a driving force for Ca^2+^ influx (Chandy et al., 2004; Nicolaou et al., 2009) to maintain T cell Ca^2+^ homeostasis (Nicolaou et al., 2009; Trebak and Kinet, 2019). Our results showed that LrB reduced IL-2 secretion in the absence of K_V_1.3 KO in Jurkat T cells, which suggests that LrB might be able to directly regulate intracellular Ca^2+^ concentration ([Ca^2+^]_i_) in Jurkat T cells. To test this possibility, we examined whether LrB affects [Ca^2+^]_i_ after Ca^2+^ stored in ER was depleted by the Ca^2+^-ATPase inhibitor CPA. After CPA exhausts Ca^2+^ in ER, CRAC channel will sense the Ca^2+^ depletion and mediate Ca^2+^ influx (Hoth and Penner, 1992; McCarl et al., 2010). We incubated the cells with 0 Ca^2+^ HBSS first and conducted calcium imaging to observe the effects of LrB on [Ca^2+^]_i_. 10 μM CPA caused a small instant [Ca^2+^]_i_ increase in Jurkat T cells. After CPA treatment for 6 min, perfusion with HBSS adding 2 mM Ca^2+^ led to a large [Ca^2+^]_i_ peak followed by a stable plateau (Figure 3E,G). Interestingly, the [Ca^2+^]_i_ reached a peak but would not go down when the cells were pretreated with 10 μM LrB in both wild type and K_V_1.3 KO groups (Figure 3F,H), which suggests that LrB might block the Ca^2+^ reuptake of the Jurkat T cells. F340/F380 ratio representing [Ca^2+^]_i_ of wild type Jurkat T cells was changed by 0.21 ± 0.024 after 2 mM Ca^2+^ perfusion, while in the K_V_1.3 KO Jurkat T cells it was changed by 0.14 ± 0.019, which is significantly smaller than the wild type group (Figure 3I). LrB pretreatment inhibited Ca^2+^ influx induced by 2 mM Ca^2+^in both the wild type (0.1748 ± 0.015) and K_V_1.3 KO Jurkat T cells (0.053 ± 0.022) (Figure 3I). Although [Ca^2+^]_i_ changes in the K_V_1.3 KO Jurkat T cells were significantly smaller than that in the wild type cells, LrB further reduced the [Ca^2+^]_i_ in the K_V_1.3 KO cells from 0.14 ± 0.019 to 0.053 ± 0.022 (Figure 3I). These results suggest that LrB inhibited Ca^2+^ influx in the absence of K_V_1.3, which is consistent with the results for IL-2 secretion.

### Inhibition of STIM1/Orai1 Current by LrB

**FIGURE 4:**
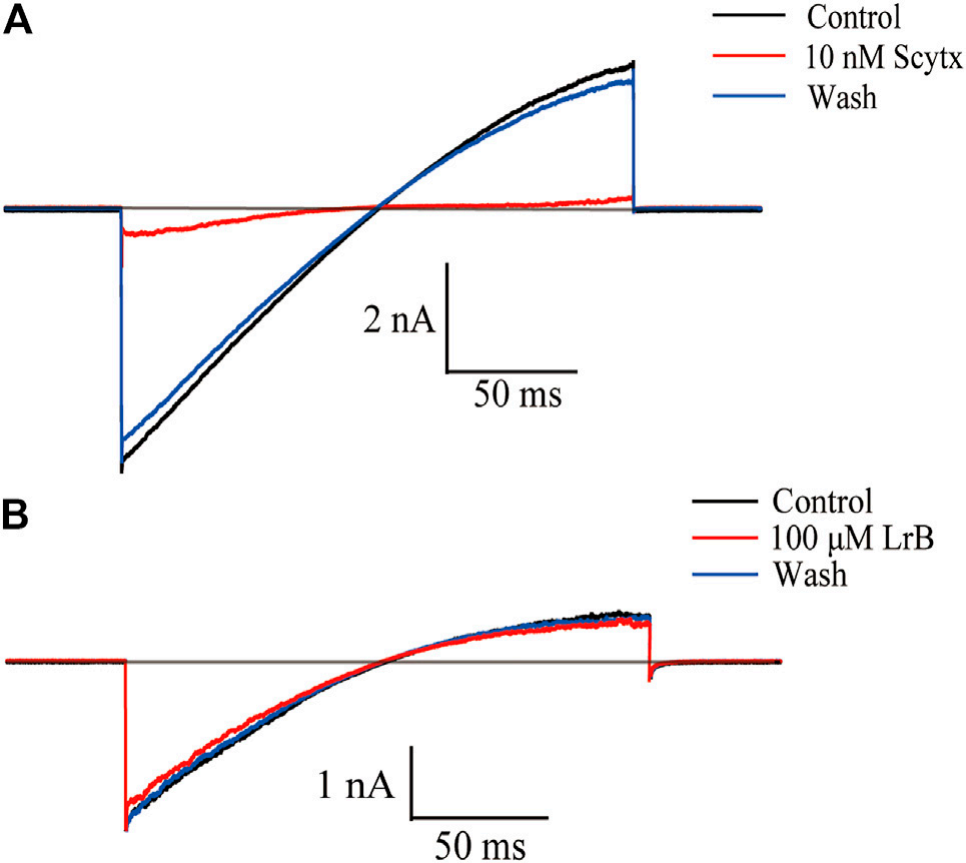
Effects of LrB on SK_Ca_ currents. **(A)** Representative whole-cell currents recorded in K_Ca_2.2 expressing HEK293T cells with or without the treatment of 10 nM Scytx. Black trace represents the baseline control current, red trace represents the currents recorded after Scytx treatment, and the blue trace is the current under external solution washing off Scytx. (*n* = 5). **(B)** Representative whole-cell currents recorded in K_Ca_2.2 expressing HEK293 T cells with and without the treatment of 100 μM LrB. Black trace represents the baseline control current, red trace represents the currents recorded after LrB treatment, and the blue one is the current under external solution washing off LrB (*n* = 5).

K_V_1.3 and K_Ca_2.2 are two main K^+^ channels expressed in Jurkat T cells and regulate membrane potential and T cell activation (Grissmer et al., 1992; Desai et al., 2000). To exclude the possibility that LrB affects SK_Ca_ to inhibit IL-2 secretion or Ca^2+^ influx in K_V_1.3 KO cells, we transfected HEK293 T cells with K_Ca_2.2 plasmids and recorded SK_Ca_ currents with or without LrB treatment. As predicted, 10 nM SK_Ca_ inhibitor Scytx (Wu et al., 2004) nearly blocked the SK_Ca_ currents (Figure 4A). In marked contrast, a relatively high concentration (100 μM) of LrB could not inhibit SK_Ca_ currents (Figure 4B), suggesting that SK_Ca_ is unlikely the mediator of LrB’s inhibition on IL-2 release and Ca^2+^ influx.

**FIGURE 5:**
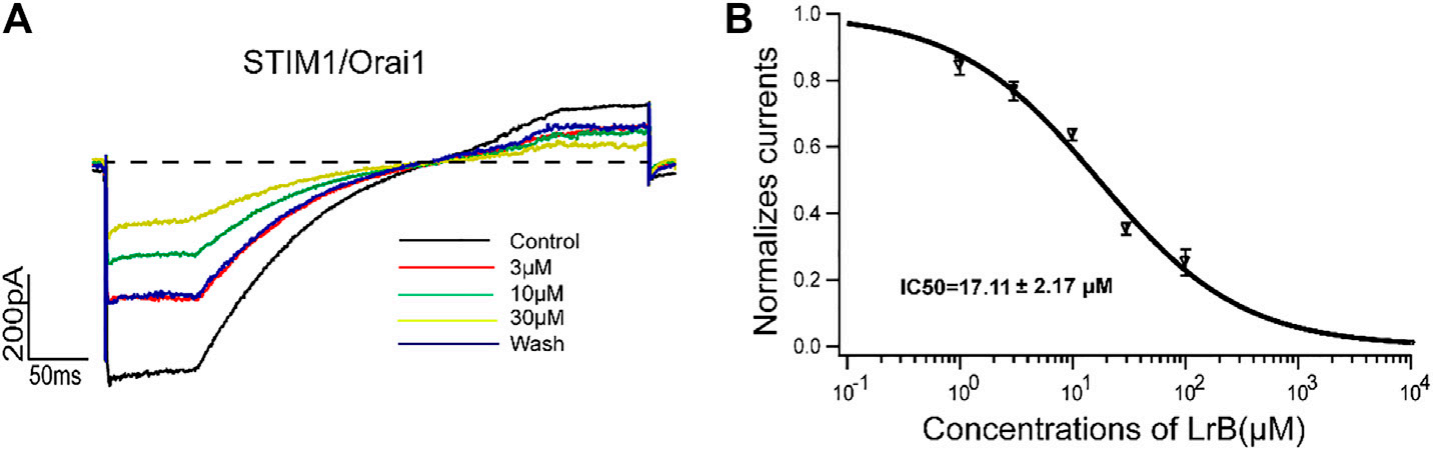
Effects of LrB on STIM1/Orai1 currents. **(A)** Representative whole-cell currents recorded in STIM1/Orai1 expressing HEK293 T cells after treatment with 3, 10, or 30 μM LrB, black trace represents the baseline control current. Red, green, and yellow traces represent the currents recorded after the treatment of 3, 10, and 30 μM LrB, respectively, and the blue trace is the current under external solution washing off LrB. **(B)** Concentration-response curve of LrB inhibition on STIM1/Orai1 currents (IC50 = 17.11 ± 2.17 μM, *n* = 5, Mean ± SEM).

Although K_V_1.3 and K_Ca_2.2 provide the driving force for Ca^2+^ influx, STIM1/Orai1 channel is the “key player” to trigger Ca^2+^ influx after ER depletion and T cell activation (McCarl et al., 2010; Trebak and Kinet, 2019). Since LrB continued to decrease Ca^2+^ influx after K_V_1.3 knockout and did not inhibit SK_Ca_, we speculated that LrB might directly inhibit STIM1/Orai1 channel and block Ca^2+^ influx in Jurkat T cells, resulting in immunosuppression. To test this possibility, we transfected the HEK293 T cells with STIM1 and Orai1 plasmids. 24 h after transfection, cells were stimulated with a ramp protocol (depolarizing the cell with a slope from the holding potential −60 to 10 mV) to record STIM1/Orai1 current. Indeed, LrB inhibited the STIM1/Orai1 current in a concentration-dependent manner. Moreover, the inhibition was partly reversed by washing off LrB in the external solution (Figure 5A). Statistics analysis with Igor Pro four Hill software showed that LrB inhibited STIM1/Orai1 current with an IC_50_ of 17.11 ± 2.17 μM (Figure 5B).

## Discussion

In this study, we used Jurkat T cell as a T cell model and applied CRISPR/Cas9 system to KO K_V_1.3 to study the effects of LrB on Ca^2+^ influx and cytokine release in the absence of K_V_1.3. We found that although KO K_V_1.3 reduced IL-2 secretion and Ca^2+^ influx after T cell activation, it did not abolish the inhibitory effect of LrB on IL-2 secretion and Ca^2+^ influx. We further demonstrated that LrB did not affect SKCa channels but directly inhibited the STIM1/Orai1 channel in heterologous cells. Our results suggest that LrB acts on multiple targets in Jurkat T cells to impact cytokine production and release. Recognizing the promiscuous property of LrB-induced inhibition of T cell function should shed new light on its immunosuppressive functions.

K_V_1.3 and SK_Ca_ channels are two predominant K channels expressed in T lymphocytes (Valle-Reyes et al., 2018; Trebak and Kinet, 2019). Both of them can regulate membrane potential and provide a driving force for Ca^2+^ influx in T cells (Cahalan and Chandy, 2009; Nicolaou et al., 2009), which subsequently modulates proliferation, activation, and apoptosis of T cells (Cahalan and Chandy, 2009; Valle-Reyes et al., 2018). It was reported that K_V_1.3 joins the immunological synapse (IS) of T cells which contacts with the antigen-presenting cell (APC) when the antigens activate TCR (Nicolaou et al., 2007; Nicolaou et al., 2009). The redistribution to IS and function of K_V_1.3 were disrupted in many immunology diseases such as systemic lupus erythematosus and rheumatic arthritis (Nicolaou et al., 2007). To investigate the mechanisms underlying K_V_1.3-mediated regulation of immune responses in diseases, numerous studies have used the K_V_1.3 KO animals, which have greatly extended our knowledge about K_V_1.3 and its functions in immune system. Initially, K_V_1.3 KO mice were found to manifest a normal phenotype with unaltered T cell proliferation and activation (Koni et al., 2003). On the other hand, the expression of Cl^−^ channels in T cells in the absence of K_V_1.3 was increased 10-fold, which is considered as a compensatory factor for the loss of K_V_1.3 to sustain the membrane potential of T cell and driving force for Ca^2+^ influx (Koni et al., 2003). Proliferation of CD4^+^ T cells and secretion of IL-17 and TNF-α were significantly decreased when EAE was induced in the K_V_1.3 KO animals compared with the wild type group (Gocke et al., 2012). These changes render the Kv1.3 KO mice resistant to EAE and support K_V_1.3 as a target to treat autoimmune diseases (Gocke et al., 2012). Another study using Th cells isolated from the K_V_1.3 KO mice expressing MOG-specific TCR (2D2-Kv1.3 KO) demonstrated that Ca^2+^ oscillation and NFATc1 activation in Th cells did not differ between K_V_1.3 KO and wild type cells after antigen stimulation, but part of the Th cells grew into a novel phenotype which is similar to regulatory T (Treg) cells (Grishkan et al., 2015). Collectively, these studies demonstrated that different types of T cells or the same cells in different stages may show divergent effects after K_V_1.3 deletion. We chose the commonly used Jurkat T cell line and applied CRISPR/Cas9 to KO K_V_1.3, which might help to reveal the effects of LrB on T cells in the absence of K_V_1.3 acutely since it may avoid the changes in Cl^−^ channel expression or other molecular compensation mechanisms during T cell development *in vivo*. This approach could be developed into a useful *in vitro* model to complement the studies using *in vivo* K_V_1.3 KO animals.

STIM1/Orai1 signaling plays pivotal roles in regulating activation, proliferation, and motility of T cells (Lewis, 2020), as reflected by the lethality of Orai1 knockout mice (Vig et al., 2008; Oh-Hora et al., 2013). Humans with Orai1 mutations also suffer from severe combined immunodeficiency (SCID) (Le Deist et al., 1995; Feske et al., 2006). STIM1/Orai1 and K_V_1.3 channels crosstalk with each other and dynamically regulate Ca^2+^ homeostasis and the downstream signaling pathways of T cells (Lioudyno et al., 2008; Nicolaou et al., 2009). It is reported that after T cell activation, STIM1/Orai1 expression is up-regulated and Ca^2+^ influx increases through a positive feedback loop (Lioudyno et al., 2008). In addition to the role of sustaining a hyperpolarization state of plasma membrane and providing a driving force for Ca^2+^ influx, K_V_1.3 anchors molecules on plasma membranes to stabilize IS and generate a sustained Ca^2+^ influx (Hanada et al., 1997; Levite et al., 2000). Our results from calcium imaging and IL-2 secretion assays showed that both Ca^2+^ influx and IL-2 secretion were reduced significantly in K_V_1.3 KO cells and the effect on IL-2 secretion was higher than that for Ca^2+^ influx. In K_V_1.3 KO cells, CRAC channels may still sense Ca^2+^ depletion in ER and mediate an instant Ca^2+^ influx when 2 mM Ca^2+^ was present in the external solution, however, this low level of Ca^2+^ increase may not be enough to form a positive feedback loop to further upregulate the downstream signaling as occurrs in the wild type cells. This may explain the discrepancy between the changes of Ca^2+^ influx and IL-2 secretion. A detailed analysis of the long-term changes of Ca^2+^ oscillations and activation of signaling molecules such as CaN and NFATc1 in K_V_1.3 KO Jurkat T cell is needed to test this possibility.

We showed that LrB significantly inhibited Ca^2+^ influx in the K_V_1.3 KO Jurkat T cell and STIM1/Orai1 currents in HEK293 T cell. Although IL-2 secretion was largely blocked by KO K_V_1.3, the remaining IL-2 was further inhibited by LrB in even at 0.01 uM concentration. This result suggests that LrB is a potent inhibitor for STIM1/Orai1 in T cells and inhibits IL-2 release in a relatively low concentration. This might be caused by the fact that other than crosslinking with K_V_1.3, STIM1/Orai1 were also closely working with other molecules to cooperatively regulate the activation of T cells (Trebak and Kinet, 2019). For instance, after the initial Ca^2+^ influx mediated by CRAC channel, mitochondria and plasma membrane calcium ATPase (PMCAs) would move close to STIM1/Orai1 channel in the IS and form a microdomain where Ca^2+^ concentration was kept in a lower level to avoid the Ca^2+^ inactivation of CRAC channel and keep a sustained Ca^2+^ elevation globally to activate Ca^2+^ signals and cytokine expression (Quintana et al., 2011). In addition, Orai1, STIM1/2, and ryanodine receptor type1 (RyR1) could also form Ca^2+^ microdomains when T cell was activated and spread Ca^2+^ increase deeper in the cell (Diercks et al., 2018). The cooperation between STIM1/Orai1 and PMCAs or RyRs may be able to explain the inhibition of Ca^2+^ reuptake by LrB in both wild type and K_V_1.3 KO Jurkat T cells. Blockade of STIM1/Orai1 by LrB might further inhibit the function of other Ca^2+^-related components such PMCAs and RyRs and block Ca^2+^ reuptake in the cytoplasm. Ca^2+^ influx through CRAC channel is also required for dynamic polymerization and depolymerization of actin and affects function and motility of the T cells (Hartzell et al., 2016; Dong et al., 2017). Therefore, CRAC channel is a highly sensitive Ca^2+^ modulator through which even a small decrease of Ca^2+^ influx could be sensed and amplified by Ca^2+^ compartmentation and cooperation with other Ca^2+^ signal regulation components. Because the numbers of CRAC channel inhibitor is limited, our finding that LrB is a potent CRAC inhibitor might provide a new direction for the development of CRAC inhibitors based on the structure of LrB, which will also facilitate the use of LrB and its derivatives as potentially highly effective medicines to treat autoimmune diseases.

## Data Availability

The original contributions presented in the study are included in the article/Supplementary Material, further inquiries can be directed to the corresponding author.
