# Supplementary material for: Loureirin B Exerts its Immunosuppressive Effects by Inhibiting STIM1/Orai1 and KV1.3 Channels
Source: Front Pharmacol. 2021 Jun 25;12:685092. doi: 10.3389/fphar.2021.685092 (PMC8268022; doi:10.3389/fphar.2021.685092)
Supplement: Supplementary file 1 [file DataSheet2.PDF]

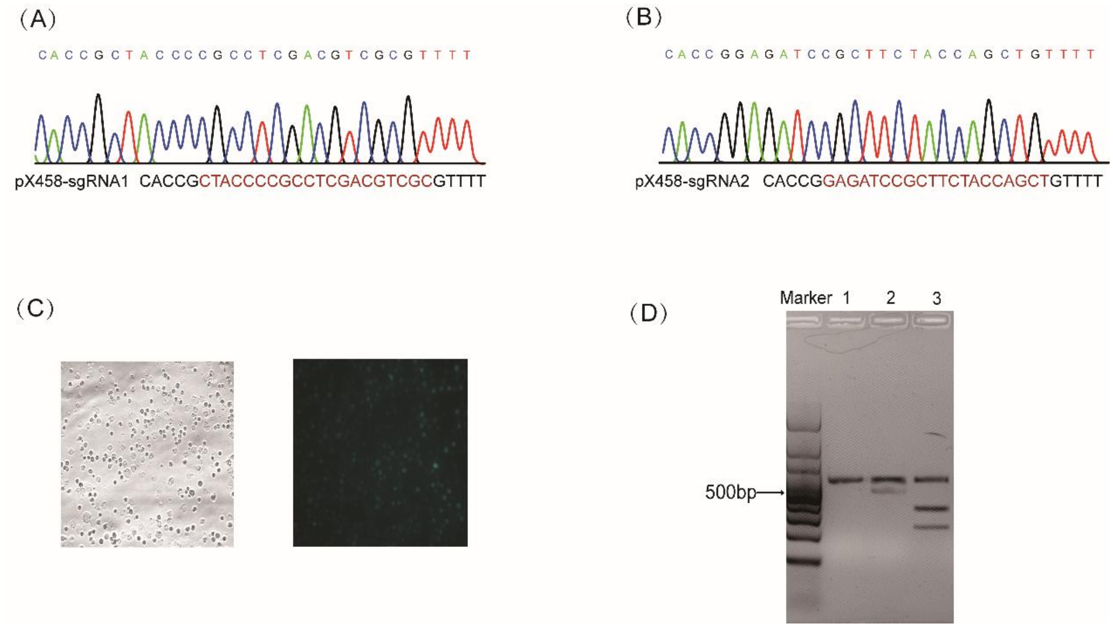

Fig.S1 Validation of pX458-sgRNA-mediated Kv1.3 KO in Jurkat T cells.

(A), (B). Sequencing of pX458-sgRNA1 (A) and pX458-sgRNA2 (B); (C). Images of cultured Jurkat T cells captured after pX458-sgRNA plasmid transfection. Left, Jurkat T cells under DIC (20×), right, Jurkat T cells under GFP stimulation (20×). (D). T7EN1 enzyme digestion of the target sequence of KCNA3. Lane 1 represents the PCR band of wildtype Jurkat T cell digested by T7EN1, lane 2 and lane 3 represents the target sequence edited by pX458-sgRNA1 and pX458-sgRNA2 separately and digested by T7EN1.
