# Supplementary material for: Loureirin B Exerts its Immunosuppressive Effects by Inhibiting STIM1/Orai1 and KV1.3 Channels
Source: Front Pharmacol. 2021 Jun 25;12:685092. doi: 10.3389/fphar.2021.685092 (PMC8268022; doi:10.3389/fphar.2021.685092)
Supplement: Supplementary file 2 [file DataSheet1.PDF]

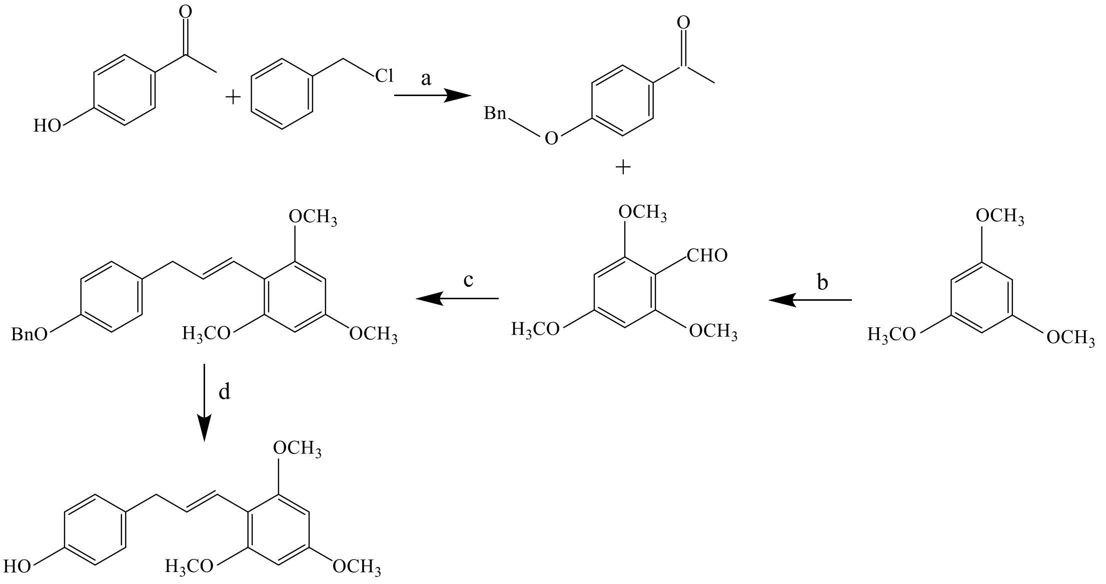

Scheme 1. Synthesis of LrB. Reagents and conditions: (a) K_2CO_3, DMF; (b) Step1: DMF, $POCl_3$, ice bath; Step2: 30 °C; (c) KOH, CH_3OH, 50 °C; (d) 10% PdC, $HCOONH_2$.
